# Data recording and use of data tools for pig health management: perspectives of stakeholders in pig farming

**DOI:** 10.3389/fvets.2024.1490770

**Published:** 2025-01-16

**Authors:** Xiao Zhou, Andrea Knörr, Beatriz Garcia Morante, Carla Correia-Gomes, Lucia Dieste Pérez, Joaquim Segalés, Marina Sibila, Carles Vilalta, Alison Burrell, Tijs Tobias, Michael Siegrist, Angela Bearth

**Affiliations:** ^1^Consumer Behavior, Department of Health Sciences and Technology, ETH Zurich, Zurich, Switzerland; ^2^Institute of Agrifood Research and Technology (IRTA), Programa de Sanitat Animal, Centre de Recerca en Sanitat Animal (CReSA, IRTA-UAB), Cerdanyola del Vallès, Spain; ^3^Unitat Mixta d'Investigació IRTA-UAB en Sanitat Animal, Centre de Recerca en Sanitat Animal (CReSA), Campus de la Universitat Autònoma de Barcelona (UAB), Barcelona, Spain; ^4^WOAH Collaborating Center for Research and Control of Emerging and Re-Emerging Pig Diseases (IRTA-CReSA), Barcelona, Spain; ^5^Animal Health Ireland, Carrick-on-Shannon, Ireland; ^6^Royal GD, Deventer, Netherlands; ^7^Departament de Sanitat i Anatomia Animals, Facultat de Veterinària, Universitat Autònoma de Barcelona (UAB), Barcelona, Spain

**Keywords:** pig farming, data tools, pig health, smart farming, sensoring

## Abstract

**Introduction:**

Data-driven strategies might combat the spreading of infectious pig disease and improve the early detection of potential pig health problems. The current study aimed to explore individual views on data recording and use of data tools for pig health management by recruiting stakeholders (*N* = 202) in Spain, Ireland, and the Netherlands.

**Methods:**

Questionnaire focused on current on-farm challenges, current status of data recording on farms, and evaluation of the two mock data tools. Particularly, “benchmarking tool” was designed to visualize individual farm’s pig mortality, targeting the management of infectious respiratory and gastrointestinal diseases; and “early-warning tool” was designed to generate an alarm through monitoring coughs in pigs, targeting the management of infectious respiratory diseases.

**Results:**

Results showed that respiratory and gastrointestinal diseases and aggressive behaviors were the most frequently mentioned health challenge and welfare challenge, respectively. Most of the data was more frequently recorded electronically than on paper. In general, the “benchmarking tool” was perceived as useful for the management of infectious respiratory and gastrointestinal diseases, and the “early-warning tool” was evaluated as useful for the management of infectious respiratory diseases. Several barriers to the perceived usefulness of these two tools were identified, such as the lack of contextual information, inconvenience of data input, limited internet access, reliance on one’s own experience and observation, technical hurdles, and mistrust of information output. The perceived usefulness of both tools was higher among highly educated participants, and those who reported being integrators and positive toward technology for disease control. Female participants and those who came from integrated farms evaluated the “early-warning tool” as more useful compared to their counterparts. The perceived usefulness of the “early-warning tool” was negatively affected by age and work experience, but positively affected by extensiveness of data recording, positive attitude toward technology, and the current use of technology.

**Discussion:**

In summary, participants showed optimistic views on the use of data tools to support their decision-making and management of infectious pig respiratory and gastrointestinal diseases. It is noteworthy that data tools should not only convey the value of data for informed decision-making but also consider stakeholders’ preconditions and needs for data tools.

## 1 Introduction

Pig production and pork export are major pillars in European agriculture, holding a great economic importance among farming sectors (1). Pig production systems, farm sizes, and management practices vary between and within EU countries, ranging from small-scale backyard production to large-scale intensive indoor production (2). To date, intensive production has become increasingly common in modern farming and has shown advantages, such as increased productivity and competitiveness, reduced impact on the environment, and enhanced quality management (3). However, it is criticized by the public for its unnaturalness, use of antimicrobials, and negative impact on animal welfare (4). Particularly, most Europeans citizens supported better protection for the welfare of farm animals (5). Aside from public perceptions, the pig industry faces substantial challenges in managing pig health (3). For instance, porcine respiratory disease complex and porcine enteric complex are among the most frequent clinical problems in pig farming, which can significantly impact production, cause economic losses, as well as affect animal welfare (6–10). The prevalence of infectious respiratory and gastrointestinal diseases is linked to multiple factors, such as infectious agent, stocking density, indoor environment, and production management (3, 11, 12), contributing to the difficulty in controlling and managing these diseases. Considering the public concerns about pig farming, the complexity of infectious disease factors, and the economic impact caused by pig diseases, monitoring and adjusting farming practices to the current situation and early detection of diseases is crucial for the pig industry (13).

To improve pig production, technologies such as monitoring systems, information management systems, and decision-support systems have been developed to utilize the available data for on-farm decision-making (14–18). Particularly, precision livestock farming (PLF) has been designed to improve livestock management by consciously monitoring animals and utilizing the data generated by sensors (17, 19). PLF allows real-time monitoring of animals with the implementation of sensors that generate data to be used by livestock farming stakeholders (19). Compared with visual observation and manual data collection, these technologies increase the quantity and consistency of the collected data, assisting stakeholders in collecting, recording, managing, and analyzing data (17, 18). Recently, data analytics and machine learning have been applied to support stakeholders’ decision-making regarding the occurrence and prevention of disease, production performance, and pig growth (20–22). For instance, routinely collected production data can be used by a syndromic surveillance system to monitor the trends of simulated outbreaks of the porcine respiratory and reproduction syndrome (PRRS) and detect signals that point to PRRS virus infection early (21). Also, coughing can be recorded and quantified by sensors, which can be further used to monitor and detect respiratory disease early with the application of artificial intelligence technologies (22).

Despite the potential that these technological developments might have for pig health and disease control, it still requires validations of these technologies under commercial farming conditions (23–25). In addition, it is crucial to address stakeholders’ concerns about the employment of technologies in pig farming (26–28). For instance, some farmers and producers expressed their worries about the cost, maintenance, user-friendliness, and lack of benefits of applying PLF (26, 27). While pig industry stakeholders’ views on data utilization technology have been investigated, on-farm preconditions and their evaluation of specific digital tools for the management of pig health, particularly in infectious respiratory and gastrointestinal diseases, remain unknown.

To support industry stakeholders’ decision-making, raw data needs to be transformed into useful information, ideally adapted to the decision makers’ information processing capacity (18, 29). For this, it is important to understand stakeholders’ needs for digital tools and evaluate which kind of information and functionalities are perceived as useful for their daily management of pig health and welfare (18). In its simplest form, information could be displayed or visualized in a dashboard for monitoring and support of data-driven decisions. Sarikaya et al. (30) summarized three functionalities or ways of using dashboards: for generating alerts based on real-time data and predefined thresholds, for benchmarking, and for visualization of information, where data is frequently updated (automatically). The chances that a tool is seen as useful and is being used by stakeholders are increased if it supports the management of or tackles the most relevant on-farm challenges such as infectious respiratory and gastrointestinal diseases in pigs which often result from multiple causes. A previous focus study by Zhou et al. (31) illustrated pig veterinarians’ needs for “benchmarking” and “early-warning” tools to support their pig health management, particularly in infectious respiratory disease and gastrointestinal diseases. Specifically, dashboards that visualize the health-relevant information (e.g., mortality) in time scale can help veterinarians to benchmark against farm’s performance and detect the potential disease risk. Also, data tools that generate alarms based on health indicators (e.g., cough counts) can contribute to the prevention and control of respiratory disease. While these functionalities are theoretically possible, based on the data available on farms, it remains to be investigated whether pig farming stakeholders (e.g., pig producers, integrators, farm managers, and farmers) would perceive such functionalities as useful to manage infectious respiratory and gastrointestinal diseases in pigs when specific tools are presented.

The present study aimed to fill the research gap with the use of an online survey among pig industry stakeholders in Spain, Ireland, and the Netherlands. Spain is the largest pig producer in the EU with most of the pig production being dominated by large-scale production and controlled by integrated companies which provide feed and veterinary services to farm owners through contracts (1, 32). Ireland contributes to a smaller degree to the European pig production, but Ireland is characterized by having one of the highest average pig herd sizes in Europe (33). The Netherlands has a relatively large pig production sector in the EU with a main orientation on pork export (34, 35). In both Ireland and the Netherlands, most pigs are raised on farms that are independently operated or family owned (1, 33).

This study was conducted within the DECIDE project,^1^ which aims at developing data-based tools to support on-farm decision making about contagious, non-EU-regulated respiratory and gastrointestinal diseases. Hence, the goal of the present study was to explore current on-farm challenges, status of data recording on farms, the preconditions for the use of data tools on pig farms and stakeholders’ needs and preferences regarding two mock-up data tools (i.e., “benchmarking tool” and “early-warning tool”). These goals translate into the following research questions (RQ):

- RQ1: What health and welfare challenges are top-of-the minds of stakeholders (i.e., integrators, farm owners, pig producers, farm managers, farm workers, and internal farm veterinarians who are directly employed by the farm or the company) from the pig industry?
- RQ2: How are health- and welfare-relevant data recorded on pig farms?
- RQ3: What are the stakeholders’ needs regarding two specific data tools for the management of infectious respiratory and gastrointestinal diseases in pigs (i.e., “benchmarking tool” designed for the management of infectious respiratory and gastrointestinal diseases and “early-warning tool” designed for the management of infectious reparatory disease)?
- RQ4: What are individual and on-farm preconditions for the perceived usefulness of these two specific data tools?

## 2 Methodology

### 2.1 Study design

The data for this study was collected between June and December 2023 via an anonymous online questionnaire. Target participants were integrators, farm owners, pig producers, farm managers, farm workers, and internal farm veterinarians from the swine farming industry in Spain, the Netherlands, and Ireland. Recruitment was handled by the project partners in the respective countries through different methods (i.e., personal contacts, mailing lists, and newsletters). The survey was screened and approved by the ethical committee of the Swiss Federal Institute of Technology in Zurich (EK 2023-N-33-A). All participants were informed about the DECIDE project and the goals of the survey at the start of the questionnaire and provided their consent to participate in this study.

### 2.2 Questionnaire

Table 1 shows scale questions and response options used in the current study. The full questionnaire can be found in the Supplementary material.

**Table 1:** An overview of scale questions and response options used in the online questionnaire.

Measure	Question	Response options (codes)
Role on the farm	What is your connection to pig farming?	*Closed-format response options:* Integrator of pig production (1) Owner of a pig farm or pig producer (2) Manager or tenant of a pig farm (3) Farm worker or employee working with the pigs (4) Internal farm veterinarian who takes care of the pig (5) production
Years of experience of working with pigs	How many years of experience do you have working with pigs?	*Open response field*
Age	Please indicate your age.	*Open response field*
Gender	Please indicate your gender.	*Closed-format response options:* Male (1) Female (2) Other (3) Prefer not to say (4)
Country of residence^1^	Please select the country that you currently live in.	*Closed-format response options:* Ireland (1) Spain (2) Netherlands (3)
Highest level of education	What is your highest level of education or degree you have completed?	*Closed-format response options:* Compulsory education (e.g., primary school, high school, leaving certificate) (1) Further education (e.g., apprenticeships, vocational school) (2) Higher education (e.g., university sector, technological sector, colleges of higher education) (3) Prefer not to say (4) Other, please specify (5)
Number of pigs	How many pigs do you currently keep?	*Open response field for each option:* Number of sows/gilts Piglets Weaners Fattening pigs Boars
Integration of pig farm	Is your farm part of a larger company/cooperation?	*Closed-format response options:* No, privately owned or independently run (1) Yes, partly integrated or contract farmed (2) Yes, fully integrated or company owned (3)
Pig health and welfare challenges	In the last year, what was most challenging for your pig’s health and for your pigs’ welfare, respectively (e.g., most difficult for you to handle)?	*Open response field for each option:* Pig health challenge Pig welfare challenge
Status quo of data recording on the farms	Please describe whether and how the following information is collected and/or recorded on your pig farm: List of 15 different types of data (i.e., pig mortality, records of therapeutic treatment, transport of pigs, clinical diagnosis from veterinarians, feed intake, information from laboratory, pig grow rate/weight, indoor temperature, pig abnormal behavior, outcome of therapeutic treatment, clinical signs, slaughterhouse information, water intake, indoor humidity, and indoor air quality)	*Closed-format response option^1^:* This information is not collected or recorded on my pig farm (1) Paper records (e.g., notebook, paper medicine book or record) (2) Manual electronic records (e.g., data management system and spreadsheet on a computer) (3) Automatic-entry electronic records (e.g., automated data recording system and data management system) (4) *Open-response field to list additional data*
Views on technology	How much do you agree or disagree the following statements about using new equipment and technology on the pig farm? I am worried that new equipment and technology will take my place in the pig farm. I do not think new equipment and technology will help me a lot to control pig disease outbreaks. I am worried that new equipment and technology will reduce my contact with the pigs’. I am confident in my ability of using new equipment and technology to facilitate daily pig health management.	*Closed-format response option:* Strongly disagree (1) Disagree (2) Neither disagree nor agree (3) Agree (4) Strongly agree (5)
Challenge of the management of infectious respiratory and gastrointestinal diseases	Compared to other pig health problems, how challenging is it to manage infectious respiratory and gastrointestinal diseases (e.g., enzootic pneumonia and porcine proliferative enteropathy)?	*Closed-format response options:* Not a challenge for me at all (1) A small challenge for me (2) A moderate challenge for me (3) A big challenge for me (4)
Perceived usefulness of “benchmarking tool”	How useful do you think this dashboard would be in helping you manage infectious respiratory and gastrointestinal diseases in pigs on your farm?	*Closed-format response options:* Not useful at all (1) Somewhat not useful (2) Undecided (3) Somewhat useful (4) Very useful (5) *Open-response field to indicate why participants did not perceive this tool as useful*
Perceived usefulness of “early-warning tool”	How useful do you think this dashboard would be in helping you to manage infectious respiratory diseases in pigs on your farm?	*Closed-format response options:* Not useful at all (1) Somewhat not useful (2) Undecided (3) Somewhat useful (4) Very useful (5) *Open-response field to indicate why participants did not perceive this tool as useful*
Current use of data tools (i.e., database management software, app or dashboard)	Do you use any database management software, app, or dashboard to collect, access and/or manage data for pig health and welfare?	*Closed-format response options:* No (1) Yes (2) Do not know (3) *Open-response field to indicate which one and list the most useful function*

^1^Responses to the measures of data recording for each type of the data were further coded as follows: 0 = “This information is not collected or recorded on my pig farm,” 1 = “Paper records (e.g., notebook, paper medicine book or record),” 2 = “Manual electronic records (e.g., data management system, spreadsheet on a computer)” or “Automatic-entry electronic records (e.g., automated data recording system, data management system).” Thus, a sum score to the responses for these 15 types of data ranges from 0 to 30.

The questionnaire was developed cooperatively by social, veterinary, and data scientists in English and was translated into Spanish and Dutch by a professional translation company. Afterwards, the translated questionnaires were reviewed and proofread by coauthors who are from Spain and the Netherlands. At the start of the questionnaire, participants could choose their preferred language version. Some socio-demographics and farm characteristics were collected from the participants. Then participants were required to answer their top challenges of pig health and welfare, the status quo of data recording on the farms, views on technology, and perceived challenge of the management of infectious respiratory and gastrointestinal diseases.

Particularly, two mock data tools were presented, including a “benchmarking tool” for the management of infectious respiratory and gastrointestinal disease in pigs and an “early-warning tool” for the management of infectious respiratory in pigs. The participants were informed that the data would be collected on their farm and that they could use the dashboard on their computer, laptop, tablet, or phone.

**Figure 1:**
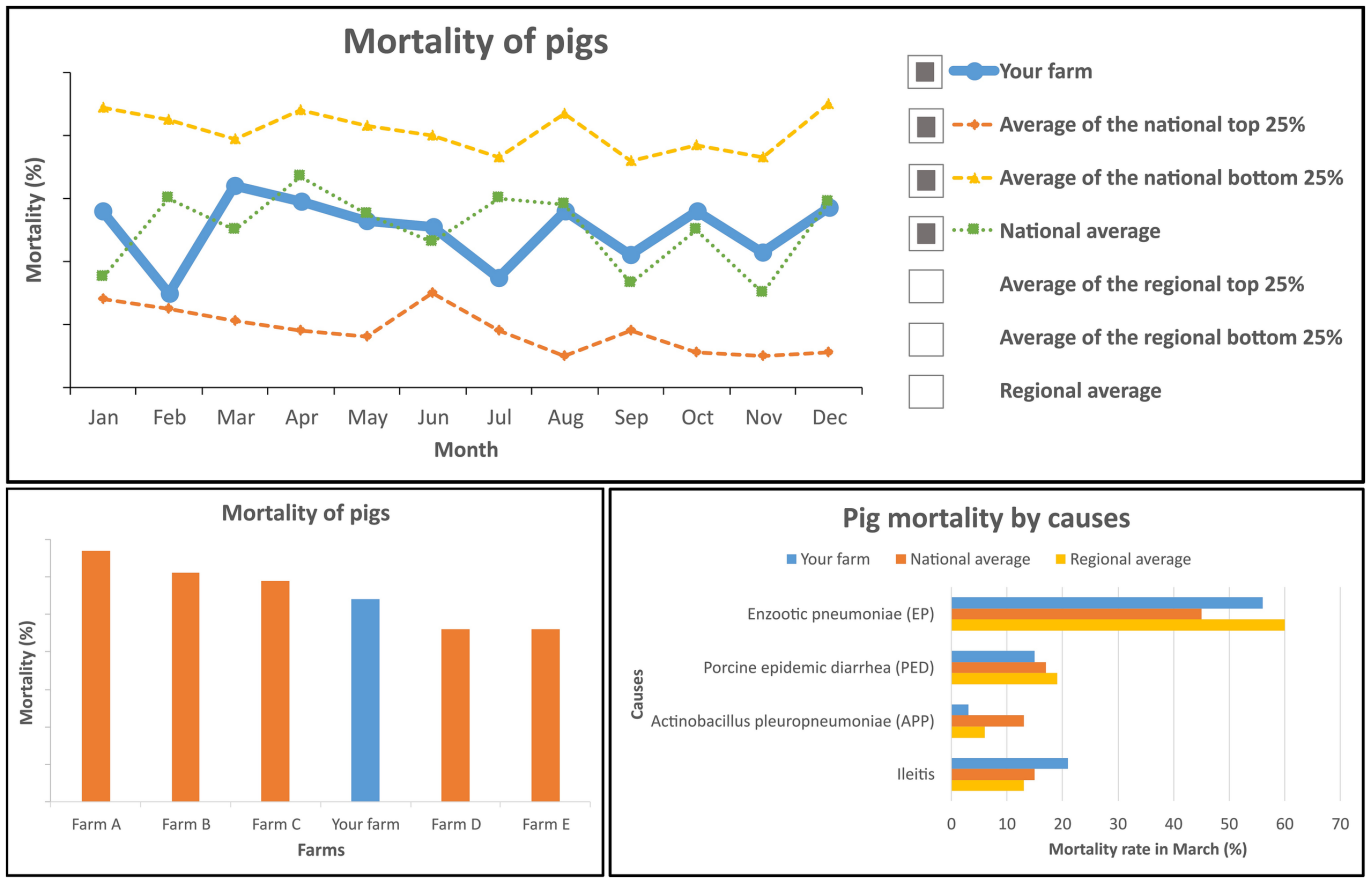
User interface image of the “benchmarking tool.”

The “benchmarking tool” was introduced with the following text and a user interface image of the tool (Figure 1): “*This dashboard displays pig mortality on your pig farm (in blue), as well as the regional and national pig mortality average. The mortality data can be shown in different time intervals (e.g., daily, weekly, monthly, and yearly). The dashboard has the following functions: (1) You can compare the mortality on your farm with that of the regional and national average levels. (2) You can compare your farm’s mortality with other pig farms nearby. (3) You can check the mortality rate by causes {e.g., % mortality caused by enzootic pneumonia (EP)/Mycoplasma hyopneumonia (Mhyo), ileitis [porcine intestinal adenopathy (PIA)]}*.”

**Figure 2:**
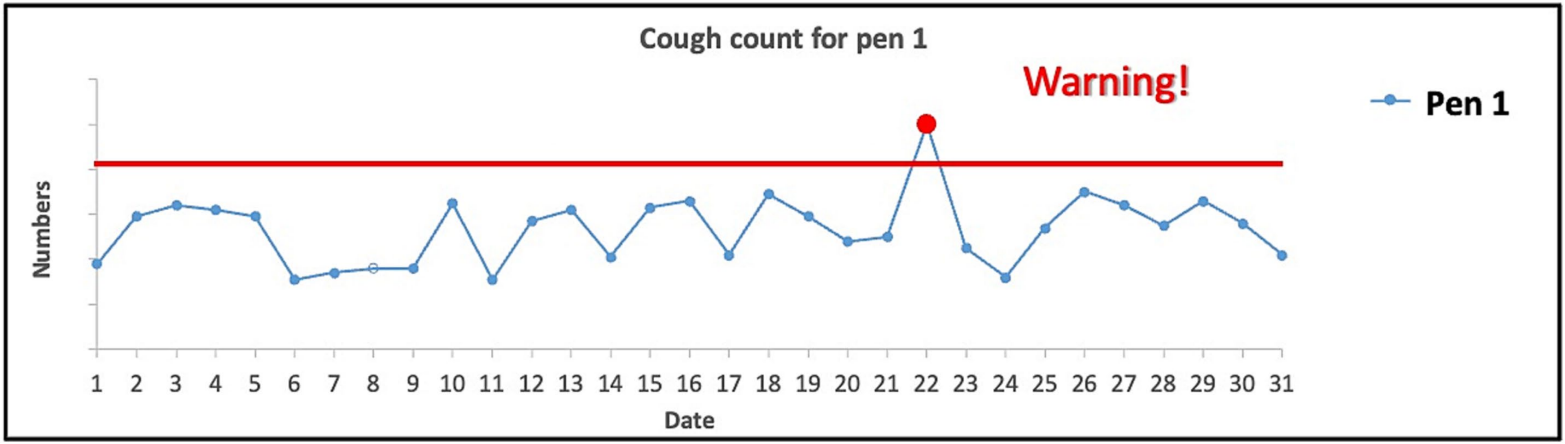
User interface image of the “early-warning tool.”

The “early-warning tool” was introduced in a similar fashion with the following text and a user interface image of the tool (Figure 2): “*Coughing is a clinical sign of respiratory diseases in pigs. Using microphones on your farm, you could collect continuous data on cough counts per pen. This dashboard can visualize cough counts per pen on your farm in real-time. When the cough count exceeds a predefined cough frequency, this dashboard can generate an alert (red line and dot). This could serve as an early warning tool to detect potential respiratory problems and facilitate control measures*.”

After the introduction of each of the two tools, the participants were asked to provide their views on the tool in a series of questions. They were asked to indicate whether this tool would be useful in helping them manage infectious respiratory and gastrointestinal diseases in pigs on their farm. Specifically, a 5-point Likert scale was used to collect participants’ response (1 = “Not useful at all,” 2 = “Somewhat not useful,” 3 = “Undecided,” 4 = “Somewhat useful” and 5 = “Very useful”). If they did not think that the tool was useful, they were asked why in an open response field.

At last, participants were asked if they use data tools to manage pig health and welfare (1 = “No,” 2 = “Yes,” 3 = “I do not know”). If yes was selected, participants were further asked to write down the name and useful functions of the data tool in open response fields.

### 2.3 Data analysis

Data cleaning and all analyses were conducted in SPSS 28.0 (36). The total number of pigs that participants currently keep was calculated by adding the number of sows/gilts, piglets, weaners, fattening pigs and boars per respondent. Four respondents reported owning or being responsible for more than a million pigs. Those responses were removed as outliers, as errors in the data entry were assumed. In addition, smaller pig farms are not the primary focus for the implementation of data tools, thus three respondents were removed as they were responsible for less than 100 pigs. The open responses regarding the pig health and welfare challenges were coded into categories. The code scheme for these categories was jointly developed by four researchers (one social scientist and three veterinary medicine researchers). The veterinary scientists sorted the responses into a flexible coding scheme. For example, the responses “PRRS outbreak” and “PRRS control” were sorted into the code PRRS. Cohen’s Kappa is an established measure to assess the level of agreement across the coders. Higher values suggest a higher agreement among the coders (37). Cohen’s Kappa ranged between 0.85 and 0.94 for health challenges and between 0.67 and 0.99 for welfare challenges, respectively. Then the social scientist resolved all conflicts in the sorting of the other three coders and summarized participants’ responses to health challenges and welfare challenges, respectively. In the rare cases where the participants provided more than one challenge in the open response field, only the first one mentioned was sorted into the coding scheme. For the status quo of data recording on the farms, a sum score was calculated by assigning scores to the response options (i.e., 0 for data that is not collected or recorded, 1 for paper records, 2 for electronic records, Table 1), signifying the availability of data on the respondent’s farm. A higher sum score signifies more extensive and digital on-farm data recording.

Table 2 summarizes the statistical methods used to address research questions in this study. Specifically, descriptive statistics for RQ1, RQ2 and RQ3 were conducted to describe frequency distributions and values of central tendencies and variance. Several bi- and multivariate analyses (i.e., Spearman correlation analysis (Spearman’s Rho), Kruskal-Wallis test (*H* test) and Mann–Whitney test (*U* test) were conducted for RQ4 to assess the relationships between sociodemographic, on-farm and individual preconditions and the perceived usefulness of data tools). Particularly, participants’ educational level was relabelled from “compulsory education,” “further education,” and “higher education” to “low education (i.e., compulsory education and further education)” and “high education.” Although of interest, comparisons across countries could not be made due to the very small sample size in the Netherlands.

**Table 2:** An overview of statistical methods used in this study.

Statistical methods	Measures	Research question (RQ)
Descriptive statistics	Socio-demographic and farm characteristic	
	Responses to pig health and welfare challenge	RQ1
	Response distribution regarding the data recorded on pig farms	RQ2
	Views on technology	
	Responses to the challenge of managing infectious respiratory and gastrointestinal diseases	
	Response to the perceived usefulness of “benchmarking tool” and “early-warning tool”	RQ3
	Responses to the current use of data tools	
Cohen’s Kappa	The level of agreement across the coders regarding the sort of participants’ responses to pig health and welfare challenges	RQ1
Spearman’s Rho	Correlations between perceived usefulness of the “benchmarking tool” and the “early-warning tool” and participants’ work experience, age, total number of pigs, challenges of the managing infectious respiratory and gastrointestinal diseases, reported extensiveness of on-farm data recording, and views on technology	RQ4
Kruskal-Wallis test	Differences in perceived usefulness of the “benchmarking tool” and the “early-warning tool” based on participants’ role on the farm and affiliation of integrated pig farm	RQ4
Mann–Whitney test	Differences in perceived usefulness of the “benchmarking tool” and the “early-warning tool” based on participants’ gender, educational level, and current use of data tools	RQ4

## 3 Results

### 3.1 Study sample and farm characteristics

A total of *N* = 285 participants took part in the survey, however, some participants dropped out over the course of the questionnaire. To retain as many responses as possible, participants were kept in the final sample if they progressed at least to the questions about the status quo of data recording on the farm. Thus, the final sample comprised *N* = 202 (*n* = 179 completed the questions of the current study, i.e., at least completed the last compulsory question regarding the current use of data tools, *n* = 23 dropped out over the course of the questionnaire) stakeholders from the pig farming industry (*n* = 151 male (75%), *n* = 49 female (24%), *n* = 2 preferred not to disclose (1%); *Mean* (*M*)_age_ = 48, *Standard Deviation* (*SD*)_age_ = 12, range_age_ = 22–80; years of experience working with pigs: *M* = 24, *SD* = 12, range = 1–50). Most participants were from Spain (*n* = 159, 79%), followed by Ireland (*n* = 33, 16%) and the Netherlands (*n* = 10, 5%). Educational level was skewed toward higher education with *n* = 135 (67%) of participants indicating higher education (e.g., university sector, technological sector, colleges of higher education), *n* = 34 (17%) further education (e.g., apprenticeship, vocational school) and *n* = 25 (12%) compulsory education (e.g., primary school, high school, leaving certificate; *n* = 8, 4% did not want to disclose).

Table 3 provides an overview of the role of the participants in pig farming and whether their farm is integrated or not. Although the sample of integrators is small, they were retained in the final sample due to their important influence on management practices on farms throughout these countries. With regards to the sizes of the farming operations that the respondents were responsible for or worked at, the following median numbers of pigs per type were observed: 1,200 sows/gilts (*n* = 140), 1,850 piglets (*n* = 126), 3,000 weaners (*n* = 127), 3,000 fattening pigs (*n* = 147) and 4 boars (*n* = 126). The following median numbers of pigs were reported by participants from Spain (*Median* = 6,405, *n* = 144), Ireland (*Median* = 7,033.5, *n* = 32) and the Netherlands (*Median* = 3,832, *n* = 9). The following median numbers were reported for partly integrated (*Median* = 11,312, *n* = 25), fully integrated farms (*Median* = 9,104, *n* = 67) and privately owned or independently run farms (*Median* = 4,851, *n* = 93). The following median numbers were reported by owners (*Median* = 4,501.5, *n* = 88), managers and tenants of a pig farm (*Median* = 10,765, *n* = 15), farm workers or employees (*Median* = 4,102, *n* = 23) and internal farm veterinarians (*Median* = 25,915, *n* = 59).

**Table 3:** Participants’ on-farm role and farm types (*N* = 202).

	Privately owned/independently run	Partly integrated/contract farmed	Fully integrated/company owned	Total
Owner/pig producer	56	11	21	88 (44%)
Internal farm veterinarian	25	13	24	62 (31%)
Farm worker or employee	10	3	11	24 (12%)
Manager/tenant	3	0	12	15 (7%)
Integrators^1^				13 (6%)
				202 (100%)

^1^The number of integrators was listed separately in Table 3 as they were not asked about the type of farms in which they work.

### 3.2 Top-of-the-mind pig health and welfare challenges

Table 4 shows an overview of all coded health and welfare challenges mentioned by the participants. Regarding the pig health challenges, the most frequently mentioned were specifically PRRS and more globally gastrointestinal diseases including diarrhea. Regarding the pig welfare challenges, the most frequently mentioned were tail biting, tail docking or ear biting, issues related to pig housing and feeding, and environmental challenges (e.g., hot weather, poor climate). Additionally, the data suggests that the participants did not strictly differentiate between health and welfare challenges. For instance, some participants regarded pig disease such as PRRS and tail biting as both health and welfare challenges.

**Table 4:** Participants’ responses to pig health and welfare challenges (*N* = 202).

Code	*n^1^*	%
Pig health challenges		
Porcine reproductive and respiratory syndrome (PRRS)	76	39%
Gastrointestinal diseases including diarrhea	32	17%
Respiratory disease (general)	19	10%
Medication use (incl. vaccines)	18	9%
Other infectious diseases (e.g., Brucellosis, Polyserositis, Leptospirosis, Erysipelas)	17	9%
Biosecurity	7	4%
Non-(primary) infectious processes (e.g., tail and ear biting, hernias, gastric ulcers)	7	3%
Locomotion disease (lameness)	6	3%
Environment and farm management	3	2%
Other/not categorizable	8	4%
Pig welfare challenges		
Tail biting, tail docking or ear biting	60	33%
Housing and feeding	41	22%
Environmental factors (e.g., climate, weather)	31	17%
Diseases	17	9%
Regulation and rules	10	5%
Medication use	3	2%
Lameness	3	2%
Biosecurity	2	1%
Stress/behavior	2	1%
Other/not categorizable	15	8%

^1^ Not all participants responded to the questions about pig health and welfare challenges (participants who did not respond to health challenges: *n* = 4; participants who did not respond to welfare challenges: *n* = 7); “No challenges” (participants who responded “no challenges” to health challenges: *n* = 5; participants who responded “no challenges” to welfare challenges: *n* = 11) was coded as missing value.

The participants provided mixed responses regarding the degree to which the management of infectious respiratory and gastrointestinal diseases posed a challenge to them (*n* = 12 did not respond to this question): About equal numbers of participants indicated that this was a small (*n* = 69, 36%) and moderate challenge (*n* = 71, 37%). Smaller numbers of participants thought that it was not a challenge for them (*n* = 35, 18%) or a big challenge for them (*n* = 15, 8%).

### 3.3 Data recording on pig farms

**Figure 3:**
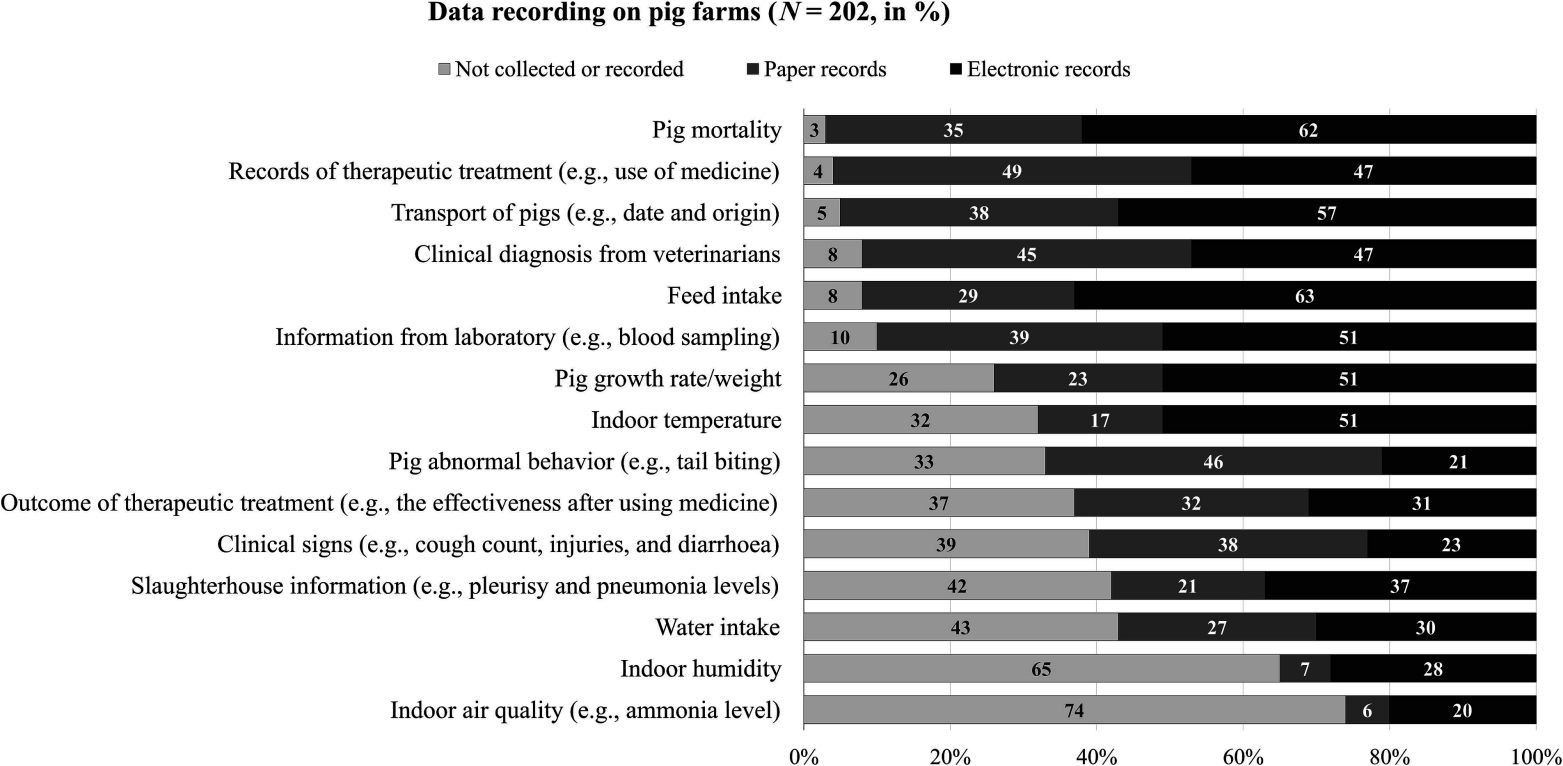
Participants’ response distributions regarding data recording on pig farms (*N* = 202, in %).

Figure 3 shows the response distributions regarding the data recorded on pig farms. Only a few participants indicated that they did not collect or record pig mortality, therapeutic treatment, transport of pigs, clinical diagnosis from veterinarians, and feed intake. Around a quarter to two fifth of participants indicated that they did not collect or record data on pig growth rate/weight, indoor temperature, abnormal behavior, outcomes of therapeutic treatment and clinical signs. Even more participants do not collect or record slaughterhouse information, water intake, indoor humidity, and indoor air quality. Overall, the participants more frequently reported electronic records (manual and automatic) than paper records, with some exceptions (i.e., therapeutic treatment, abnormal behavior, outcomes of therapeutic treatment and clinical signs). There was a significant correlation between total number of pigs and the extent of on-farm data recording, *r_s_* = 0.21, *p* = 0.004, *n* = 185 (*n* = 17 missing responses to the number of pigs). There was no significant difference in the extent of on-farm data recording for privately run, partly or fully integrated farms revealed by the Kruskal-Wallis test, *H*(2) = 2.2, *p* = 0.339.

The participants listed a variety of additional health- and welfare-related data that is collected on their farms in the open response field (e.g., routine vaccines, welfare observations, new challenges emerging in a herd, biosecurity measures, sow/boar productivity, visitor registration). Some participants also mentioned how they recorded these additional data: with chalk on the wall on the farm (i.e., abnormal behavior, peculiarities), on the animal with brand spray (i.e., abnormal behavior), on paper (i.e., routine vaccines and animal welfare monitoring), on pictures and videos (i.e., behavioral patterns, tail biting as proof for the need to dock, lameness, signs of disease), and electronically (i.e., farm visits by veterinarians and biosecurity survey).

A total of *n* = 82 participants (46%) indicated that they already used a database management software, app or dashboard to collect, access and/or manage data for pig health and welfare, while *n* = 90 (50%) did not and *n* = 7 (4%) did not know (*n* = 23 did not respond to this question). Most frequently, established national or international tools specific for (pig) farming were mentioned (*n* = 31; e.g., WinPig, Nedap Velos, SoundTalks, Animal Ireland Pig HealthCheck), some participants mentioned spreadsheet programs (*n* = 5) or tools that they had developed themselves (*n* = 6); *n* = 40 did not specify the tool that they used. The participants highlighted various functions, namely monitoring specific data (*n* = 22), early detection (*n* = 4) and data visualization (*n* = 4); *n* = 52 did not mention any specific function.

**Figure 4:**
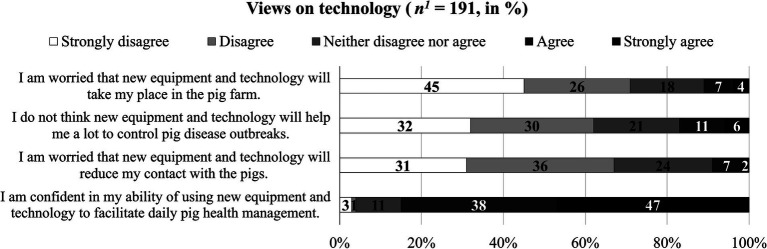
Participants’ views on technology (*n* = 191, in %). ^1^Not all participants responded to questions about the views on technology (*n* = 11).

Figure 4 shows the responses regarding the views on technology. Overall, the participants exhibited optimistic views toward new equipment and technology on their farms. A majority of 71, 62, and 67% disagreed or strongly disagreed to be worried about the technology taking their place, the lack of usefulness regarding the technology for pig disease control, and the reduced contact with the pigs caused by technology adoption, respectively. Similarly, a majority of 85% agreed or strongly agreed that they were confident in their own abilities to use new equipment and technology to facilitate daily pig health management.

### 3.4 Stakeholders’ needs regarding two specific data tools

Both mock data tools for the management of infectious respiratory and gastrointestinal diseases in pigs were rated as reasonably useful (“benchmarking tool”: *M* = 4.0, *SD* = 1.0; “early-warning tool”: *M* = 4.0, *SD* = 1.1). There was a significant positive correlation between the perceived usefulness of the “benchmarking tool” and the “early-warning tool” (*r_s_* = 0.28, *p* < 0.001, *n* = 181).

The participants who thought that the “benchmarking tool” was not useful (*n* = 18, 10%) indicated that contextual information was missing (*n* = 7; e.g., differentiation of the cause of mortality, about the type of other farms, about statistical significance of differences in mortality), lack of confidence in the validity of the data (*n* = 2), unwillingness to input this data or use a data tool, linked to their reliance on their own experience (*n* = 4); one participant indicated that the veterinarians had already used a similar system; four participants did not indicate a reason. The participants who thought that the “early-warning tool” was not useful (*n* = 17, 9%) indicated the lack of internet access (*n* = 1), the unwanted substitution of individual observations with data (*n* = 2), expected technical hurdles (*n* = 2), lack of confidence in the validity of the data (*n* = 1) and unwillingness to install sensors (*n* = 1); *n* = 10 did not indicate a reason.

### 3.5 Individual and on-farm preconditions for the perceived usefulness of data tools

Table 5 shows the correlation analyses between perceived usefulness of the two mock data tools and continuous sociodemographic and pig farm variables, on-farm challenges of the management of infectious respiratory and gastrointestinal diseases, the extensiveness of on-farm data recording and individual views on technology as independent variables. The perceived usefulness of the “benchmarking tool” was not significantly related to any of the variables except its negative correlation with participants’ worries about the technology for pig disease control. The perceived usefulness of the “early-warning tool,” however, was negatively correlated with age, years of experience of working with pigs, participants’ worries about the lack of usefulness regarding the technology for pig disease control, and their worries about the reduced contact with pigs (i.e., the older and more experienced the participant was, and the more worried about the usefulness and reduced contact with pigs, the less useful they perceived the early-warning tool to be). Additionally, the perceived usefulness of the “early-warning tool” was positively linked to the extensiveness of data recording and participants’ confidence in their own ability to use technology (i.e., more extensive data recording and higher confidence was associated with higher perceived benefit).

**Table 5:** Correlations between perceived usefulness of the “benchmarking tool” and the “early-warning tool” and participants’ sociodemographic, on-farm preconditions and views on technology.

	*r_s_* Benchmarking tool	*r_s_* Early-warning tool
	*n = 186*	*n = 181*
Years of experience of working with pigs	−0.04	−0.22**
Age	0.03	−0.18*
Total number of pigs	0.06	0.09
Challenge of the management of infectious respiratory and gastrointestinal diseases	−0.03	0.03
Extensiveness of on-farm data recording	0.08	0.16*
Views on technology		
*Worry about technology taking their place*	−0.09	−0.09
*Worry about the lack of usefulness regarding the technology for disease control*	−0.35**	−0.44**
*Worry about reduced contact with pigs*	−0.06	−0.15*
*Confidence in own ability to use technology*	0.12	0.26**

r_s_, Spearman’s correlation coefficient; *p < 0.05; **p < 0.01; participants who did not respond to the perceived usefulness of “benchmarking tool”: *n* = 16; participants who did not respond to the perceived usefulness of “early-warning tool”: *n* = 21.

Table 6 shows the results regarding the differences in perceived usefulness across categorical sociodemographic and farm variables and if participants indicated that they already used a database management software, app or dashboard to collect, access and/or manage data for pig health and welfare. Regarding the “benchmarking tool,” integrators and participants with higher education expressed higher perceived usefulness than participants in other roles or with lower education. Regarding the “early-warning tool,” integrators, farm workers and internal farm veterinarians expressed higher perceived usefulness than owners, managers and tenants. Also, women and participants with higher education expressed higher perceived usefulness of the “early-warning tool” than men and participants with lower education. Moreover, the “early-warning tool” was perceived as more useful in partly- or fully integrated pig farms than in privately-owned or independently run pig farms. Participants who reported that they already used data tools for pig health and welfare management showed higher perceived usefulness of the “early-warning tool” than those who did not use data tools.

**Table 6:** Differences in perceived usefulness of the “benchmarking tool” and the “early-warning tool” based on participants’ sociodemographic, on-farm preconditions and current use of data tools.

	*M* (*SD*)	Test statistics
Benchmarking tool		
Role on the farm		*H* (4) = 15.3, *p* = 0.004
*Integrator (n = 12)*	4.8 (0.5)^a^	
*Internal farm veterinarian (n = 58)*	4.2 (0.7)^b^	
*Owner of a pig farm*/*pig producer (n = 83)*	3.8 (1.1)^c^	
*Manager*/*tenant of a pig farm (n = 11)*	3.7 (1.2)^b,c^	
*Farm worker or employee (n = 22)*	3.7 (1.4)^b,c^	
Integration of pig farm		*H* (2) = 2.8, *p* = 0.242
*Privately-owned*/*independently run (n = 88)*	3.8 (1.0)^a^	
*Partly integrated (n = 25)*	4.2 (0.7)^a^	
*Fully integrated (n = 61)*	3.9 (1.2)^a^	
Gender		*U* = 3200.5, *p* = 0.886
*Male (n = 138)*	4.0 (1.0)^a^	
*Female (n = 47)*	3.9 (1.1)^a^	
Highest level of education		*U* = 2381.5, *p* = 0.001
*Low^1^ (n = 53)*	3.6 (1.1)^b^	
*High^2^ (n = 126)*	4.1 (1.0)^a^	
Current use of data tools^3^		*U* = 3418.0, *p* = 0.371
*No (n = 90)*	3.9 (1.2) ^a^	
*Yes (n = 82)*	4.1 (0.9) ^a^	
Early-warning tool		
Role on the farm		*H* (4) = 17.3, *p* = 0.002
*Integrator (n = 12)*	4.6 (0.7)^a^	
*Farm worker or employee (n = 21)*	4.3 (1.1)^a^	
*Internal farm veterinarian (n = 57)*	4.2 (1.0)^a^	
*Owner of a pig farm/pig producer (n = 81)*	3.7 (1.1)^b^	
*Manager/tenant of a pig farm (n = 10)*	3.6 (1.0)^b^	
Integration of pig farm		*H* (2) = 14.0, *p* < 0.001
*Partly integrated (n = 86)*	4.5 (0.8)^a^	
*Fully integrated (n = 25)*	4.1 (1.0)^a^	
*Privately-owned/independently run (n = 58)*	3.7 (1.1)^b^	
Gender		*U* = 2316.0, *p* = 0.017
*Man (n = 136)*	3.9 (1.1)^b^	
*Woman (n = 44)*	4.3 (0.9)^a^	
Highest level of education		*U* = 2488.5, *p* = 0.023
*Low (n = 51)*	3.7 (1.2)^b^	
*High (n = 123)*	4.2 (1.0)^a^	
Current use of data tools^3^		*U* = 3010.5, *p* = 0.027
*No (n = 90)*	3.9 (1.1)^b^	
*Yes (n = 82)*	4.2 (0.9)^a^	

M, Mean; SD, Standard deviation; H, Kruskal-Wallis test; U, Mann–Whitney test; Different superscript letters indicate significant differences according to post hoc tests (*p* < 0.05); ^1^compulsory education and further education; ^2^ higher education; ^3^*n* = 7 participants were excluded as they responded “I do not know” to the question about the current use of data tool for pig health and welfare management.

## 4 Discussion

### 4.1 General insights

In terms of on-farm challenges, pig farming stakeholders did not strictly differ between health and welfare challenges. Although these two concepts are different, animal welfare refers to “the physical and mental state of an animal in relation to the conditions in which it lives and dies” (38), for instance the “freedom from pain, injury or disease” (39), which might explain the observed similarities in some stakeholders’ responses to the challenges of managing pig health and welfare. Particularly, respiratory diseases (specifically PRRS) and gastrointestinal diseases were seen as the most challenging health problems by pig farming stakeholders. To tackle these challenges, a previous qualitative study by Zhou et al. (31) has illustrated pig veterinarians’ needs for tools that utilize currently available or easily collectible data, turn it into useful information to monitor diseases or recommend preventative or mitigating actions to control the spread of diseases. Indeed, the value of using data tools such as syndromic surveillance tools to monitor and predict the trends of diseases (e.g., PRRS) in pigs based on the recorded data on farms has been uncovered by previous research (21, 40). With the assistance of artificial intelligence and machine learning, trends and patterns in data can be recognized to support stakeholders’ disease diagnoses and guide treatments (41). Furthermore, pig welfare problems such as tail biting, stock density, feeding conversion, dietary roughage, and house environmental factors were mentioned as the primary concerns by pig farming stakeholders, which has been highlighted as important aspects of pig welfare assessment in previous studies (3, 42, 43). Nowadays, some sensor technologies at the pen (e.g., tools monitoring the pen environment or detecting coughing) and individual (e.g., electronic feeders and weigh scales) levels are commercially available for farmers to monitor pig behavior and manage pig health. However, well-validated and farm-applicable technologies that can be used by stakeholders to detect abnormal behaviors and specific diseases have not yet been available and transitioned to commercial products (23–25, 44, 45).

Our study finds that most participants already recorded data about pig health, mortality, and transport of pigs. This aligns with the emphasis on recording these data by the EU regulation (46) with the aim of enhancing traceability and control of animal disease. The extensiveness of on-farm data recording is associated with the farm size, indicating that digital tools might be more accessible to and easy to implement in larger farms that already record a lot of data in electronic format. Consistently, the benefits of automated data collection using technologies to improve efficiency in dairy cattle management has been mentioned by farmers in the previous study (47). Our study further shows that a wide variety of data is recorded on farms through various means (e.g., paper records, pictures and videos or chalk on the wall). For one thing, some participants might lack a data tool that conveniently supports their collection and recording of these types of data. For another, many factors may hinder participants from adopting technologies for automated data collection such as their worries about data reliability, high investment, required skills of using technologies, compatibility, and network connectivity of technologies (47, 48). Still, it requires further research to reveal and meet stakeholders’ needs for specific data recording. If recording, processing, and synthesis of different types of data can be integrated in one data tool that is user-friendly, reliable, and cost-effective, stakeholders could benefit from using digital tools for data utilization and data communication (24, 31, 47, 49).

Two data tool functionalities were investigated in more detail in this study: Data visualization and benchmarking in dashboards and early detection of respiratory disease by monitoring the frequency of pig coughs. A positive correlation between the perceived usefulness of the “benchmarking tool” and the “early-warning tool” might indicate that participants who deemed one tool useful were likely to also deem the other tool as useful and vice versa. It is noteworthy that several factors were found to be negatively associated with the perceived usefulness of these tools. For instance, the lack of contextual information in the mortality dashboard was criticized by seven participants. This finding aligns with previous qualitative studies (28, 48) that additional information such as texts recordings about changes in farm management practices and the time of the day might be helpful for farmers to improve their performances (28, 48). Still, it remains an unsolved challenge that contextual information (e.g., reason for mortality and environmental context) is difficult to collect and link to mortality information via data tools. Our findings suggest, however, that a tool that offers this would be preferred by the stakeholders more than a tool that simply displays mortality. Indeed, stakeholders in the current study mentioned that the “benchmarking tool” lacking the function of differentiating the mortality cause was useless. Furthermore, the current study as well as existing literature found other technical barriers that tool developers need to consider such as accessibility (e.g., using a data tool that requires constant internet access in remote areas) (50), trustworthiness (e.g., of the data or predictions, false positive or negative alarms) (19, 48, 51, 52), and comprehensibility (e.g., availability of training material, visualizations and analyses that match stakeholders’ skills in data analysis and interpretation) (48, 50).

Additionally, several individual and on-farm preconditions were found to be significantly associated with participants’ perceived usefulness of these two mock tools. For instance, participants with a higher educational level perceived both mock tools to be more useful than those who reported a lower educational level. This is consistent with previous studies that showed positive effects of a higher education level on farmers’ acceptance of new technologies (53, 54). Decisions in integrated pig farming are usually taken by integrators simultaneously on multiple farms (32), thus, integrators might perceive the two mock tools as more useful to support the management of infectious respiratory and gastrointestinal diseases. Notable differences can be found when examining the factors affecting the participants’ evaluations of “benchmarking tool” and “early-warning tool,” respectively. Age, gender, years of experience of working with pigs, extensiveness of on-farm data recording, individual confidence in using technology, and concern about the reduced contact with pigs and farm type were significantly associated with the perceived usefulness of the “early-warning tool,” but not with the perceived usefulness of the “benchmarking tool.” According to a previous focus group study by Zhou et al. (31), pig veterinarians mentioned the common use of software applications for visualizing and identifying abnormal data to support pig farming stakeholders’ decision-making in pig health management. Thus, the functionalities of “benchmarking tool” might have been widely accepted by participants. Furthermore, the “benchmarking tool” additionally visualizes the pig mortality on neighbor farms, which uniquely contributes to the benchmarking functions against other farmers that the “early-warning tool” has not achieved. Compared to the “benchmarking tool,” the “early-warning tool” as a relatively new technology might trigger more uncertainties and concerns about the functions and accuracy of this tool that generates alerts for detecting respiratory diseases (19, 51, 52). For instance, the accuracy of the alert generated by the “early-warning tool” might be affected by multiple factors such as the quality of data, environmental factors, and the applied algorithm for disease prediction (23, 55), lacking a validation to clearly confirm the accuracy of early warning by linking the measures from a sensor with the assessment of pig conditions (25). In the current study, the “benchmarking tool” visualizes the mortality data that is collected on farms, which may provide more transparent, reliable, and easily interpretable information than the “early-warning tool” that generated the alert via complex data process. Previous studies illustrated that the uncertainties of innovative technology and the lack of trust in technology capabilities and robustness might impact farmers’ adoption of technology in livestock farming (48, 56, 57). This might potentially explain why the perceived usefulness of the “benchmarking tool” varied less than the perceived usefulness of the “early-warning tool.” Meanwhile, this might also account for why stakeholders who already use data tools perceived the “early-warning tool” as more useful than those who did not have data tools, as they might have more confidence in using technologies and express more trust in the technology output (48, 56, 57).

Age and years of experience of working with pigs were negatively correlated with participants’ perceived usefulness of “early-warning tool,” which conforms with previous studies showing that older farmers and those who had longer work experience were less accepting of the new technology in livestock farming than younger and less experienced farmers (58, 59). Farmers’ experiences and observations play an important role in their decision-making (27, 28), thus, stakeholders with longer work experience might adhere more to their routine practice of managing pig health, e.g., recognizing abnormal pig behaviors by on-site observation instead of adopting new technologies (58). Notably, new technology might threaten farmers’ beliefs and values regarding animal keeping (60), which might account for the relatively low perceived usefulness of the “early-warning tool” among participants, who reported less confidence but expressed more concerns about reduced contact with pigs regarding the use of technologies. It is relevant to point out that farm employees and internal veterinarians also rated a higher usefulness of the “early-warning tool” than farm owners and managers. Indeed, although human observations of pigs are important for detecting potential health problems, it is impractical and laborious to manually monitor pigs especially on large-scale farms, requiring technological assistance through providing consistent and quantitative data (47, 61). Furthermore, an “early-warning tool” may help integrators to remotely monitor pig health status and detect the potential risk of respiratory disease at an early stage, as integrators have a lower frequency of inspecting pigs compared to farmers. With the application of the “early-warning tool,” integrators might enhance their efficiency of risk communication and decision-making between integrators and other stakeholders such as the veterinarians and contract farmers. This might explain why stakeholders linked to partially or fully integrated farms rated the “early-warning tool” as more useful than those who come from privately owned or independently run farms. Additionally, it is unsurprising that participants who reported extensive data recording on their farms and already used data tools for pig health and welfare management evaluated the “early-warning tool” as more useful compared to their counterparts. More extensive data recording in an electronic format and current use of data tools might drive stakeholders to embrace new technologies to get more value from the collected data (62, 63).

### 4.2 Implications and recommendations

The current study offers important recommendations for tool developers who aim to support stakeholders’ decision-making through data utilization. Generally, pig industry stakeholders in Spain, Ireland, and the Netherlands evaluated the two mock data tools for managing infectious respiratory and gastrointestinal diseases positively. Particularly, for dashboards, skills in handling, analyzing and visualizing data might be needed. This requires a two-pronged approach of first, a careful consideration of the dashboard’s most likely user group and second, a user-friendly, intuitive, easy-to-access and easily readable interface (23). While a previous study has uncovered the importance of training and support from tool developers for technology adoption, some farmers still felt that the trainings they received were insufficient to fully master the use of technology (48). Thus, providing easily accessible instructions for users are needed and the advantages of data tools need to be convincingly demonstrated, e.g., through the validation of the tool’s performances by considering the stakeholders’ involvement, as this might increase stakeholders’ confidence in using data tools (40, 48, 50). Moreover, it is noteworthy to develop the tool that aligns with users’ needs by considering the variety of farm situations (48). Furthermore, integrators, young pig farming stakeholders, and large, integrated pig farms may be the primary target for tool developers. Lastly, several barriers need to be addressed when employing data tools on farms. For instance, ensuring a good and stable internet connection on farms (50), improving procedures of collecting and recording data, such as contextual data, and maintenance in technological equipment (23, 50).

### 4.3 Limitations and future studies

Several limitations of the current study should be discussed. Firstly, despite extensive efforts to recruit a heterogeneous group of stakeholders in pig farming, a comparably small sample size was reached, particularly in Ireland and the Netherlands. Participants may not completely represent the populations of pig farming stakeholders in their respective countries.^2^ It is probably due to the various recruitment methods used in different countries (64), incentives (65), distrust of researchers (66, 67), and heavy workload (66, 67). In hindsight, a more personal approach (e.g., on-farm interviews or focus groups) might have garnered more responses but would have required substantially more resources than this online survey. Moreover, future studies could consider including major pig producing countries outside of Europe, such as China and the United States (68), to compare stakeholders’ views of technologies across countries. Secondly, although the current “benchmarking tool” was assumed to have the ability to visualize mortality data from neighbor farms, in reality, data might be more likely to be aggregated at the regional or country level to ensure anonymity (40). It is possible that such an aggregation would reduce the perceived usefulness of the tool but might reduce privacy concerns. Future research and efforts are needed to explore industry stakeholders’ preferred way of data sharing, which might contribute to the development of a surveillance system that can be used for the early detection and control of diseases. Lastly, the current study uncovers factors associated with stakeholders’ perceived usefulness of two mock tools, but how the realistic data tool impacts stakeholders’ decision-making in certain circumstances remains unknown. In the future, pilot tests are needed to improve the usability and usefulness of data tools by evaluating stakeholders’ real-time and real-world interactions with the specific functions of data tools.

## 5 Conclusion

Being aided by technologies, stakeholders can efficiently and effectively utilize data to support their decision-making and improve their management of pig health. The current study paints an optimistic picture regarding the openness of the participants toward data recording, utilization and toward data tools for managing pig health. A “benchmarking tool” and an “early-warning tool” could be useful for pig farming stakeholders to manage respiratory and gastrointestinal diseases. Importantly, the success of using data tools to support informed decision-making should not only showcase the value of data semantics but also take stakeholders’ preconditions and their needs into consideration.

## Data Availability

The raw data supporting the conclusions of this article will be made available by the authors, without undue reservation.

## Footnotes

^1^ https://decideproject.eu

^2^In Spain, the total number of pig holdings was 85,197, of which 47,557 holdings were registered as intensive farms (according to the registration status as of January 1, 2024) (69); In Ireland, the number of pig holdings in 2021 was 1,693, of which 387 holdings with more than 100 pigs were registered (70); In Netherlands, the total number of pig holdings in 2022 was 3,273 (71). It is important to note that some pig farming stakeholders such as integrators, producers, and farmers may own or manage more than one farm.
