# Supplementary material for: Data recording and use of data tools for pig health management: perspectives of stakeholders in pig farming
Source: Front Vet Sci. 2025 Jan 16;11:1490770. doi: 10.3389/fvets.2024.1490770 (PMC11782995; doi:10.3389/fvets.2024.1490770)
Supplement: Supplementary file 1 [file Data_Sheet_1.PDF]

What is your connection to pig farming?

- Integrator of pig production
- Owner of a pig farm/pig producer
- Manager/Tenant of a pig farm
- Farm worker or employee working with the pigs (does not include e.g., advisor, veterinarian, and feed supplier)
- Internal farm veterinarian who takes care of the pig production (does not include e.g., advisor, veterinarian, and feed supplier)

How many years of experience do you have working with pigs?

Please indicate your age.

Please indicate your gender

- ○ Male
- ○ Female
- ○ Other
- ○ Prefer not to say

Please select the country that you currently live in.

- ○ Ireland
- ○ Spain
- ○ Netherlands

What is your highest level of education or degree you have completed?

- ○ Compulsory education (e.g., primary school, high school, and leaving certificate)
- ○ Further education (e.g., apprenticeships, vocational school)
- ○ Higher education (e.g., university sector, technological sector, colleges of higher education)
- ○ Prefer not to say
- ○ Other, please specify ________

How many pigs do you currently keep?

Fill out all that apply

Number of sows/gilts (breeding, dry, and farrowing) ☐

Number of piglets (up to 3/4 weeks) ☐

Number of weaner (3/4 -10 weeks) ☐

Number of fattening pigs ☐

Number of boars ☐

Is your farm part of a larger company/cooperation?

- ○ No, privately owned or independently run.
- ○ Yes, partly integrated or contract farmed.
- ○ Yes, fully integrated or company owned.

In the last year, what was most challenging for your pigs' health and for your pigs' welfare, respectively (e.g., most difficult for you to handle)?

Pig health challenge	
Pig welfare challenge	

Please describe whether and how the following information is collected and/or recorded on your pig farm:

	This information is not collected or recorded on my farm	Paper records (e.g., notebook, paper medicine book or record)	Manual-entry electronic records (e.g., data management system and spreadsheet on a computer)	Automatic-entry electronic records (e.g., automated data recording system and data management system)
Feed intake	○	○	○	○
Water intake	○	○	○	○
Pig growth rate/weight	○	○	○	○
Indoor humidity	○	○	○	○
Indoor temperature	○	○	○	○
Indoor air quality (e.g., ammonia level)	○	○	○	○
Clinical diagnosis from veterinarians	○	○	○	○
Information from laboratory (e.g., blood sampling, tissue sampling, pathology, antimicrobial susceptibility test)	○	○	○	○
Records of therapeutic treatment (e.g., injections, vaccination, use of medicine)	○	○	○	○
Outcome of therapeutic treatment (e.g., the effectiveness	○	○	○	○

after using medicine)				
Pig mortality	○	○	○	○
Slaughterhouse information (e.g., pleurisy, pneumonia levels)	○	○	○	○
Clinical signs (e.g., cough count, injuries, diarrhoea)	○	○	○	○
Pig abnormal behavior (e.g., tail biting)	○	○	○	○
Transport of pigs (e.g., date and origin)	○	○	○	○

Do you collect and record any other health/welfare related information on your pig farm? Please specify it and how you collect it and/or record it.

This question is optional.

[ ]

How much do you agree or disagree the following statements about using new equipment and technology on the pig farm?

	Strongly disagree	Disagree	Neither disagree nor agree	Agree	Strongly agree
I am worried that new equipment and technology will take my place in the pig farm.	○	○	○	○	○
I do not think new equipment and technology will help me a lot to control pig disease outbreaks.	○	○	○	○	○
I am worried that new equipment and technology will reduce my contact with the pigs.	○	○	○	○	○
I am confident in my ability of using new equipment and technology to facilitate daily pig health management.	○	○	○	○	○

In the following questions, we will focus on your daily work regarding infectious respiratory and gastrointestinal diseases in pigs (e.g., enzootic pneumonia (EP)/mycoplasma hyopneumonia (Mhyo), ileitis (porcine intestinal adenopathy (PIA))).

Compared to other pig health problems, how challenging is it to manage infectious respiratory and gastrointestinal diseases?

- ○ Not a challenge for me at all.
- ○ A small challenge for me.
- ○ A moderate challenge for me.
- ○ A big challenge for me.

In the following questions, we will introduce different mock dashboards that could be used to manage infectious respiratory and gastrointestinal diseases in pigs on your farm. These dashboards are based on the data collected and recorded on your farm and you can use the dashboard via computer, laptop, tablet or phone.

We would like to ask your opinions about the dashboard functions, their usefulness, and your willingness to collect and record your data.

This dashboard displays pig mortality on your pig farm (in blue), as well as the regional and national pig mortality average. The mortality data can be shown in different time intervals (e.g., daily, weekly, monthly, and yearly). The dashboard has the following functions:

- You can compare the mortality on your fam with that of the regional and national average levels.
- You can compare your farm's mortality with other pig farms nearby.
- You can check the mortality rate by causes (eg., % mortality caused by enzootic pneumonia (EP)/ mycoplasma hyopneumonia (Mhyo), ileitis (porcine intestinal adenopathy (PIA))).

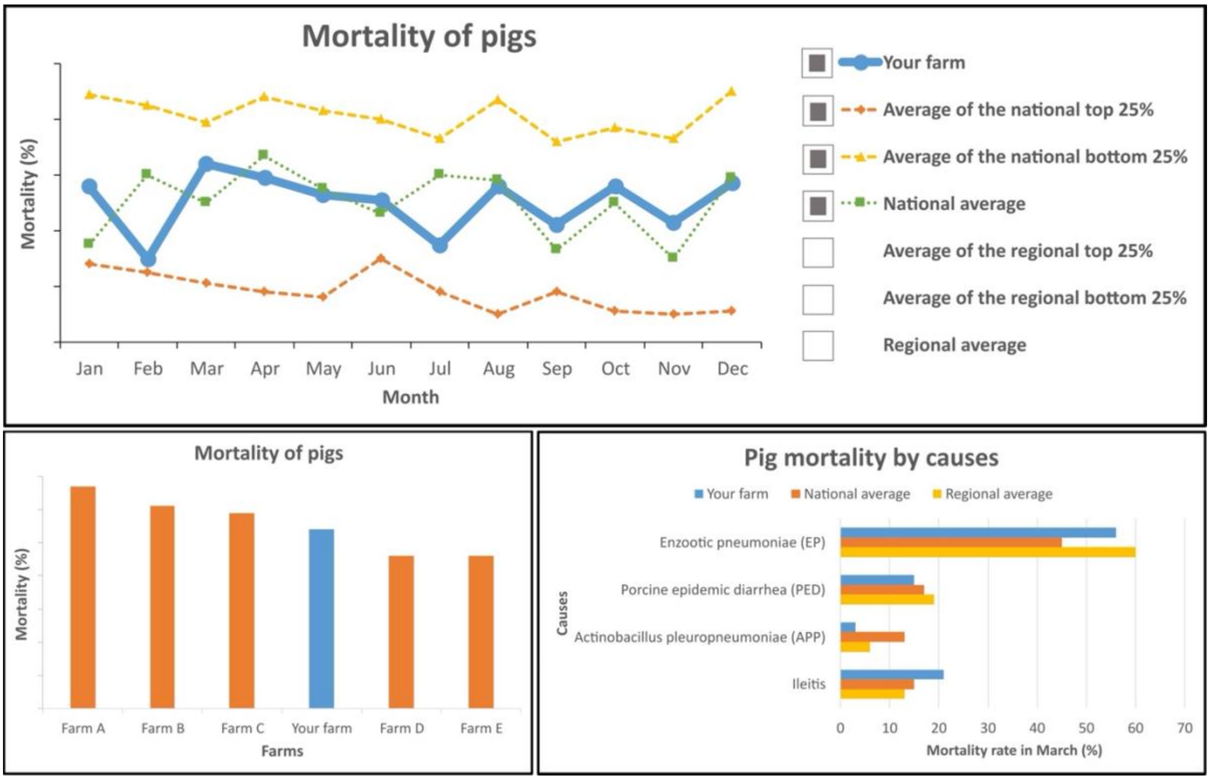

How useful do you think this dashboard would be in helping you manage <u>infectious respiratory and gastrointestinal diseases</u> in pigs on your farm?

- ○ Not useful at all
- ○ Somewhat not useful
- ○ Undecided
- ○ Somewhat useful
- ○ Very useful

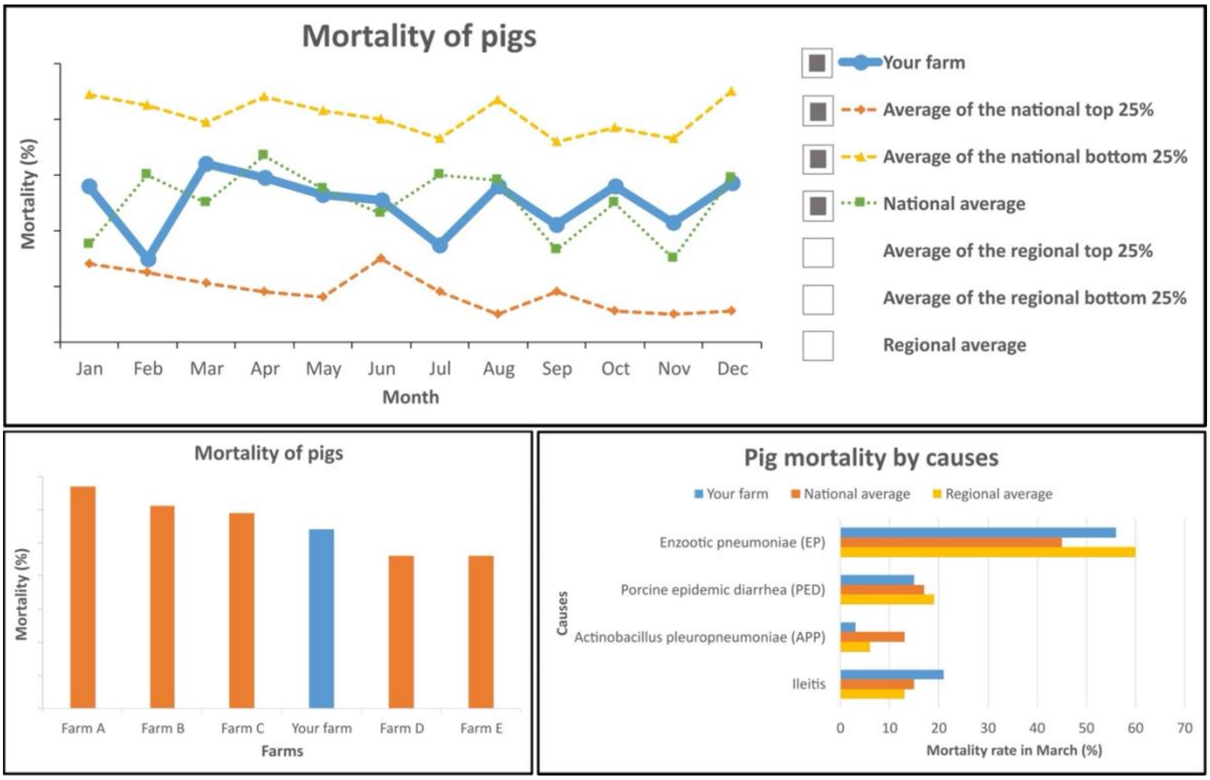

Can you explain why this dashboard is not useful for you to manage infectious respiratory and gastrointestinal diseases in pigs on your farm?

(Note: Participants were asked to answer this question only if they evaluated the "benchmarking tool" as not useful)

This question is optional.

Coughing is a clinical sign of respiratory diseases in pigs. Using microphones on your farm you could collect continuous data on cough counts per pen. This dashboard can visualise cough counts per pen on your farm in real time. When the cough count exceeds a pre-defined cough frequency, this dashboard can generate an alert (red line and dot). This could serve as an early warning tool to detect potential respiratory problems and facilitate control measures.

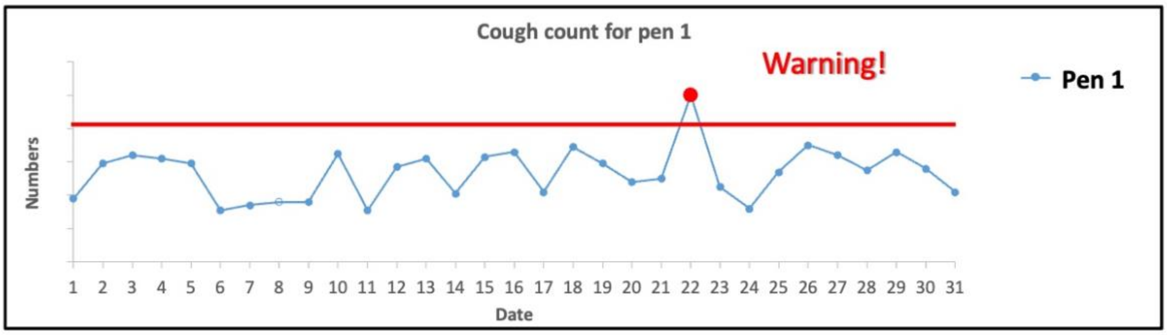

How useful do you think this dashboard would be helping you to <u>manage infectious respiratory diseases</u> in pigs on your farm?

- ○ Not useful at all
- ○ Somewhat not useful
- ○ Undecided
- ○ Somewhat useful
- ○ Very useful

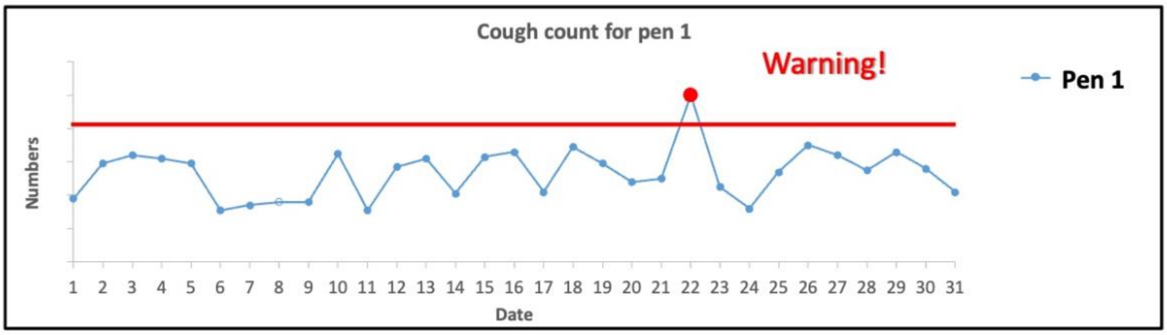

Can you explain why this dashboard is not useful for your to manage pig infectious respiratory diseases?

(Note: Participants were asked to answer this question only if they evaluated the "early-warning tool" as not useful)

This question is optional

In the following questions, we would like to know your experience of using technology to manage pig health and welfare.

Do you use any database management software, app, or dashboard to collect, access, and/or manage data for pig health and welfare?

- ○ No
- ○ Yes
- ○ I do not know

Which database management software, app, or dashboard are you using? Could you write down the name?

The question is optional

Could you please describe the most useful fuction and how you use it for pig health and /or welfare management?

The question is optional
